# Supplementary material for: Methods for discovering genomic loci exhibiting complex patterns of differential methylation
Source: BMC Bioinformatics. 2017 Sep 18;18:416. doi: 10.1186/s12859-017-1836-0 (PMC5604413; doi:10.1186/s12859-017-1836-0)
Supplement: Supplementary file 1 — Supplemental.pdf - supplementary materials. Description of simulation studies and parameter details; Figure S1: Boxplot of locus widths for each model/context; Figure S2: Profiles of cytosine methylation in additional mutants for identified differentially methylated loci; Figure S3: Profiles of model abundance across the genome; Table S1: Numbers of loci associated with models of differential methylation. (PDF 3164 kb) [file 12859_2017_1836_MOESM1_ESM.pdf]

METHODS

Methods for discovering genomic loci exhibiting complex patterns of differential methylation; Supplementary Materials

Thomas J Hardcastle

Correspondence: tjh48@cam.ac.uk
Department of Plant Sciences, University of Cambridge, Downing Street, CB2 3EA Cambridge, UK
Full list of author information is available at the end of the article

Simulation studies

Data are simulated using WGBSSuite version 0.4, modified to include the effect of non-conversion rates by introducing a binomially distributed error in the number of methylated cytosines reported at each site. Plant-representative parameters were estimated by applying the `analyse_WGBS.R` script provided by WGBSSuite to the first million CpG cytosines on chromosome 1 of a single wild-type sample and the *dcl2/3/4* triple mutant in the Stroud *et al*[**?**] dataset. Data were then simulated for one hundred thousand cytosines using the following command, where XXX and YYY are placeholders for the number of samples in each replicate group and the magnitude of difference in the proportion of methylation respectively.

```
Rscript simulate_WGBS.R 100000 0.851721358101034 0.0158503393648432
  +0.2 0.2 14.064221221 14.064221221 XXX 2 YYY 0.5
  +0.0267845702643185,0.00339225464276114 plant_simulation binomial
  +0.237414948538599,1e-15 0.0577772070946612,0.34
```

Version 0.4.4 of methylSig, 1.14 of methylKit, 1.10.0 of BSmooth and 3.4.2 of MethPipe were used to analyse these data using default settings for all libraries. Versions 2.9.5 of segmentSeq and 2.8.0 of baySeq were used to implement the approach described here.

References

. Stroud, H., Greenberg, M.C., Feng, S., Bernatavichute, Y., Jacobsen, S.: Comprehensive Analysis of Silencing Mutants Reveals Complex Regulation of the Arabidopsis Methylome. Cell **152**(1), 352–364 (2013). doi:10.1016/j.cell.2012.10.054

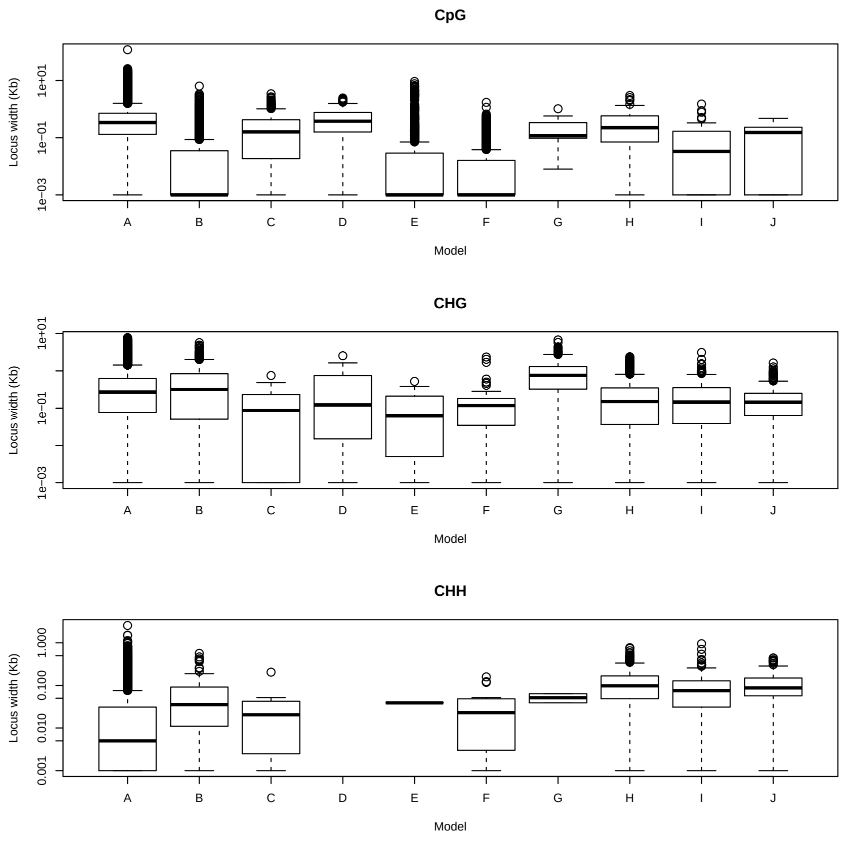

Figure S1 Boxplot of locus widths for each model/context.

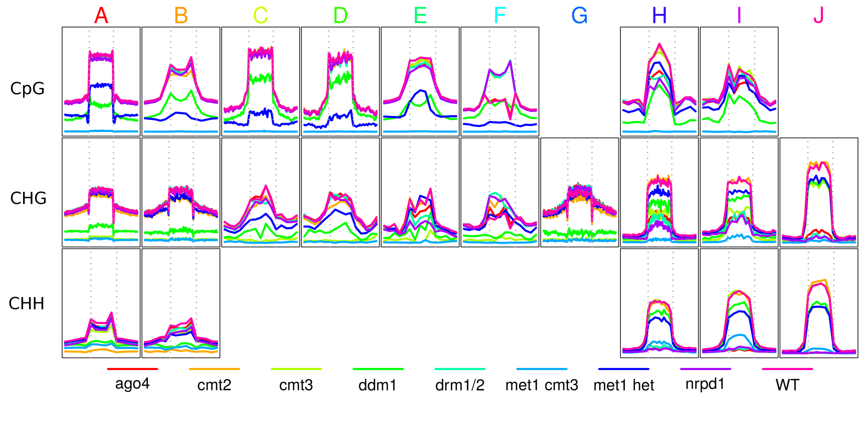

Figure S2 Profiles of cytosine methylation in additional mutants for identified differentially methylated loci. Average methylation profiles in the *ago4*, *cmt2*, *cmt3*, *ddm1*, *drm1/2*, *met1 cmt3*, *met1 het*, *nrpd1*, and wild-type (WT) samples from Stroud *et al*, across the methylation loci (and the surrounding 4Kb) identified for each model/context with an FDR of 5%. Profiles are shown for those model/context combinations in which at least 20 loci at this FDR could be identified.

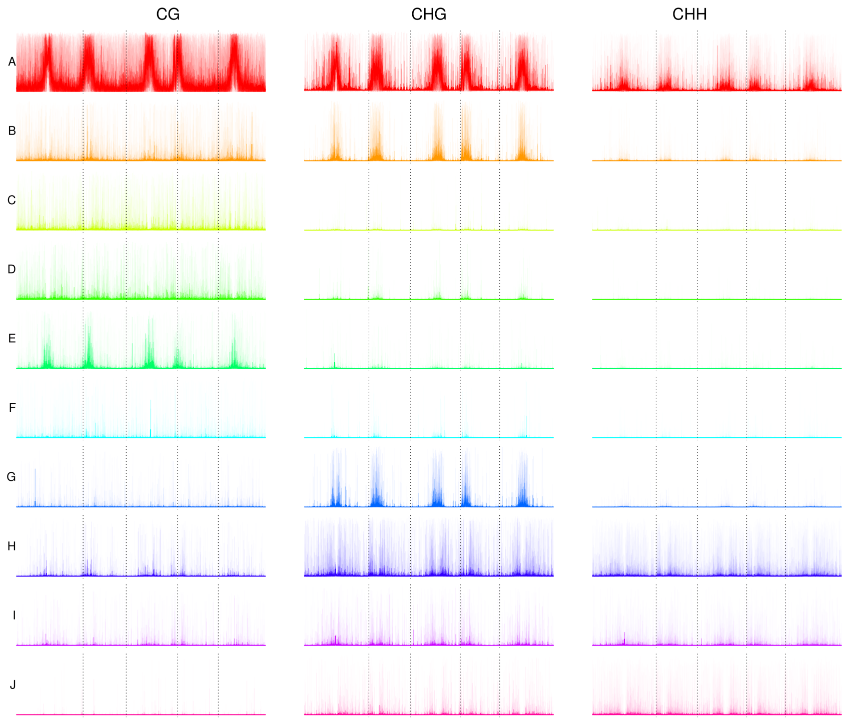

Figure S3 Profiles of model abundance across the genome. Model localisations across the genome; the average posterior likelihood observed at each base within travelling windows of 101, 1001, 10001 and 100001 bases is computed and plotted for each model and methylation context. The transparency of the plotted value increases with window size such that the larger windows have a reduced effect on the plotted values.

Table S1 Numbers of loci associated with models of differential methylation.

**Model ID**	**Model definition**	**Estimated number of loci**	**Number of identified loci (FDR < 0.05)**
A	{WT,dcl2/3/4,dcl2/4,dcl3,dcl2,dcl4}	CpG: 67365<br>CHG: 53313<br>CHH: 138593	CpG: 17607<br>CHG: 9310<br>CHH: 30058
B	{WT,dcl2/3/4,dcl2/4,dcl3,dcl4}>{dcl2}	CpG: 10769<br>CHG: 5459<br>CHH: 4686	CpG: 3587<br>CHG: 996<br>CHH: 137
C	{WT}>{dcl2/3/4,dcl2/4,dcl3,dcl2,dcl4}	CpG: 8624<br>CHG: 692<br>CHH: 2290	CpG: 119<br>CHG: 2<br>CHH: 4
D	{WT,dcl2/3/4,dcl2/4,dcl2,dcl4}>{dcl3}	CpG: 6989<br>CHG: 644<br>CHH: 1383	CpG: 1499<br>CHG: 27<br>CHH: 3
E	{WT,dcl2/3/4,dcl2/4,dcl3,dcl2}>{dcl4}	CpG: 6906<br>CHG: 631<br>CHH: 1306	CpG: 877<br>CHG: 41<br>CHH: 9
F	{dcl3}>{WT,dcl2/3/4,dcl2/4,dcl2,dcl4}	CpG: 6131<br>CHG: 866<br>CHH: 1527	CpG: 73<br>CHG: 1<br>CHH: 1
G	{WT,dcl2/3/4,dcl2/4,dcl4}>{dcl3,dcl2}	CpG: 5167<br>CHG: 654<br>CHH: 1672	CpG: 74<br>CHG: 24<br>CHH: 3
H	{dcl2/3/4,dcl2/4,dcl3,dcl2,dcl4}>{WT}	CpG: 3665<br>CHG: 797<br>CHH: 614	CpG: 1890<br>CHG: 43<br>CHH: 20
I	{WT,dcl2/3/4,dcl2/4,dcl3}>{dcl2,dcl4}	CpG: 2532<br>CHG: 738<br>CHH: 855	CpG: 281<br>CHG: 28<br>CHH: 1
J	{WT,dcl2/3/4,dcl2/4}>{dcl3,dcl2,dcl4}	CpG: 2365<br>CHG: 331<br>CHH: 307	CpG: 175<br>CHG: 1<br>CHH: 1
K	{WT,dcl2/4,dcl2,dcl4}>{dcl2/3/4,dcl3}	CpG: 2208<br>CHG: 8384<br>CHH: 13657	CpG: 62<br>CHG: 2314<br>CHH: 542
L	{WT,dcl2/3/4,dcl2/4,dcl2}>{dcl3,dcl4}	CpG: 1564<br>CHG: 229<br>CHH: 462	CpG: 79<br>CHG: 7<br>CHH: 1
M	{WT,dcl2/3/4,dcl2/4,dcl3}>{dcl4}>{dcl2}	CpG: 968<br>CHG: 9<br>CHH: 15	CpG: 218<br>CHG: 1<br>CHH: 1
N	{WT,dcl2/4,dcl3,dcl2}>{dcl2/3/4}>{dcl4}	CpG: 596<br>CHG: 7<br>CHH: 21	CpG: 92<br>CHG: 1<br>CHH: 1
O	{WT,dcl2/3/4,dcl3,dcl2}>{dcl2/4}>{dcl4}	CpG: 435<br>CHG: 2<br>CHH: 5	CpG: 62<br>CHG: 1<br>CHH: 1
P	{WT,dcl3,dcl2,dcl4}>{dcl2/4}>{dcl2/3/4}	CpG: 306<br>CHG: 12<br>CHH: 42	CpG: 74<br>CHG: 1<br>CHH: 1
Q	{WT,dcl3,dcl2,dcl4}>{dcl2/3/4}>{dcl2/4}	CpG: 301<br>CHG: 5<br>CHH: 14	CpG: 75<br>CHG: 1<br>CHH: 1
R	{dcl2/4}>{WT,dcl2/3/4,dcl3,dcl2,dcl4}	CpG: 2623<br>CHG: 3351<br>CHH: 4726	CpG: 7<br>CHG: 153<br>CHH: 1
S	{WT,dcl2/4,dcl3,dcl2,dcl4}>{dcl2/3/4}	CpG: 2298<br>CHG: 3220<br>CHH: 6387	CpG: 47<br>CHG: 360<br>CHH: 255
T	{dcl2/3/4,dcl2/4,dcl3,dcl4}>{WT,dcl2}	CpG: 1661<br>CHG: 2745<br>CHH: 1219	CpG: 16<br>CHG: 337<br>CHH: 3
U	{WT,dcl2/4,dcl2,dcl4}>{dcl3}>{dcl2/3/4}	CpG: 207<br>CHG: 1746<br>CHH: 5435	CpG: 10<br>CHG: 308<br>CHH: 307
V	{WT,dcl2/4,dcl4}>{dcl2/3/4,dcl3,dcl2}	CpG: 1719<br>CHG: 1315<br>CHH: 2831	CpG: 12<br>CHG: 63<br>CHH: 37
W	{dcl2/3/4,dcl2/4,dcl3}>{WT,dcl2,dcl4}	CpG: 2189<br>CHG: 1260<br>CHH: 606	CpG: 35<br>CHG: 54<br>CHH: 1
X	{WT,dcl2/3/4,dcl3,dcl2,dcl4}>{dcl2/4}	CpG: 1237<br>CHG: 757<br>CHH: 1886	CpG: 51<br>CHG: 79<br>CHH: 3
